# Chicago Medical Society

**Published:** 1889-04

**Authors:** 


### CHICAGO MEDICAL SOCIETY.

*Stated Meeting, January 21.*

THE PRESIDENT IN THE CHAIR.

DR. CHAS. WARRINGTON EARLE read a paper entitled:

### THE CONTAGIOUSNESS OF DIPHTHERIA, AND ITS MUNICIPAL CONTROL.

With us there has arisen the question sometimes whether a given case was diphtheria—follicular tonsilitis—tonsilitis without deposit, or simple ulcerative stomatitis. We have sometimes criticised physicians who have called a pharyngitis with a few white spots, diphtheria. In view, however, of the fact that some of these very mild cases have proven markedly contagious, it is a question with me as to whether physicians who call and treat a mild case as diphtheria, have not in the main been correct. My conversion to this procedure was brought about as follows: In 1878, I was attending a family consisting of four girls and one boy. The female children had sore throats, characterized by redness, some pain and a few white spots. It was diagnosticated as follicular tonsilitis, and the parents were informed that there was absolutely no danger. In the course of a few days the boy was taken with the same symptoms, which rapidly became more alarming. General infection in the course of two days took place, and death resulted. I have always thought that if I had diagnosticated diphtheria in the first patients, treated it as such and sent the boy away from the infected locality, he might be alive to-day. This, with other cases, has changed my diagnosis and practice entirely.

About six years ago I was treating a case. It was a mild one, and recovered rapidly. During the course of treatment a relative of the family, with two children, was journeying from the east to their new home in Dakota, and stopped for twelve hours in this infected house. Neither the mother or children were in the room with the mild case I was treating—indeed, not on the same floor of the house. They remained a single night in a remote part of the house, and in the morning resumed their journey. In one week after arriving in Dakota, one of the children sickened and died; in another week the remaining child died, and the mother barely escaped death from the same disease.

Although the contagiousness of this disease has been recognized for two hundred and fifty years, we at this day find members of our profession denying it, and refusing the greatest safeguard to not only their own families, but to the public at large, in casting their influence against isolation and other means to prevent the spread of the disease. The authorities, almost without exception, are a unit in regard to its contagiousness.

In this connection there are many questions which might with profit be discussed, such as the time of incubation, the indentity or non-indentity of croup and diptheria. But its contagiousness, in my judgment, is of the greatest importance. One would think, from our discussions, that the chief aim of the practitioner of to-day was to tube or perform tracheotomy. The object paramount, in my judgment, should be to prevent the disease, which, in large measure, can be accomplished if we are a unit as regards our opinions of contagiousness, isolation and disinfection.

In view of the mortality from this dread scourge, a hundred fold more than from small-pox, I see no reason why our local board should not have this matter in charge, investigating each home invaded by this disease, and seeing to it that what is known regarding disinfection and isolation is rigidly enforced.

Dr. Oscar C. De Wolf: I should like to put a pretty broad interrogation point after the assertion that pseudo-membranous croup and diphtheria are identical diseases. A few years ago I asked the opinion of the profession of this city on that point. I received about 500 replies, 60 per cent. of them assenting to this identity, and I have no doubt to-day that if a consensus of the professional opinion of this country and Europe were taken more than 60 per cent. would assent to the assertion that they are identical diseases, and yet, sir, I cannot escape from my own conviction—an opinion which will not, and ought not, to have very much weight in this society, yet I cannot escape from the conviction that there is a wide divergence between pseudo-membranous laryngitis—inflammatory laryngitis pure and simple, and epidemic diphtheria pure and simple. There is a vast divergence, it seems to me, in the condition of the patient passing through the disease; the one a local inflammatory condition, the other a true toxemia. And from the etiological stand-point, which must be the stand-point of preventive medicine, which must be the stand-point taken by the sanitarian who would be of use to the community which he serves, there is a still greater divergence. The one a local inflammatory condition, and the individual passing through it standing in the same relation to the public as a source of danger, as though he were suffering from an inflammatory rheumatism, no more, no less—two diseases produced by similar conditions. The other a true specific infection; the introduction of a germ which reproduces itself in the organism to a limitless extent, a germ which lies at the basis as a cause of this the most atrocious malady which afflicts the child class on the face of this earth to-day. A veritable pestilential disease, which should be classed with other pestilential diseases, differing, perhaps, from them in epidemic manifestations, or degree of diffusibility throughout a neighborhood, yet essentially of the same character as scarlet fever, small-pox, cholera or yellow fever. It was, perhaps, a mistake, at least it has led to mistake, that those eminent men, Simon and Farr, spoke of diphtheria as a filth-disease. It has misled the popular understanding; it has misled professional understanding. The profession and the people have been hunting for the cause of diphtheria in filth of domicile and surrounding, and they have neglected that prime necessity to which the orator has referred to-night, that prime factor in the case, personal intercourse, and the infection which it carries. It is not true that any amount of filth, or heat, or moisture, or any combination of these, can produce the specific germ of diphtheria any more than manuring a field will produce a crop of corn. It is possible that filth, and heat, and moisture in a domicile and surrounding it increases the mortality of this disease; though that I am somewhat disposed to doubt, from recent experience.

Now let me say a single word touching the municipal control of this disease. I will follow the line of thought of the orator in what I have to say, and it stands in about this way. We have a communicable disease infecting a house, or epidemic manifestations of that disease in the neighborhood; its diffusion throughout that neighborhood depends entirely (following the suggestion of the orator) upon the facilities of personal intercourse; which I believe. At a previous meeting of this society a resolution was introduced, at my request, suggesting an expression of opinion from this society touching the propriety of placarding that house. That resolution, much to my regret, was tabled. I will tell you why I regret it. The card upon the house indicates the professional understanding of the danger connected with the appearance of this disease in a neighborhood. It says to the family within the house, you are afflicted with one of the gravest diseases which can afflict your household, and you have become a source of danger to the neighborhood which surrounds you. It says to the passer-by, flee from that house as from a pestilence. That is the professional recognition of danger connected with that dwelling. The card on the house is a popular educator, leading the understanding of the neighborhood, of the people, up to the plane of the professional recognition of the danger. Two or three gentlemen have called upon me during the past fortnight to say they would like to see that card, but it was an annoyance to families, and the family charging the annoyance to the physician who reported it, says, Dr. A. is a mean man; we will discharge him and have Dr. B., who won't report it. I do not say that that may have induced this society to have tabled the resolution, because to admit that is to impeach the profession in the position in which it should stand before the community. One gentleman says, we will lose our patronage. I detest that word patronage, sir, in connection with our profession. I detest the word patron. You have patients, my dear sir; patients who love and respect you; *but you have no patrons.* You have patients who trust you to relieve them from their physical suffering, but the moment they feel that your neighbor is more competent to relieve them than yourself, it is their duty to themselves to go to your neighbor, and you would be the first man to hasten them. The more we get away from the idea of patronage and patrons the more we elevate our profession out of the odor and away from the methods and manners of hucksters. If a family objects to the card, it is the duty of the physician to say to them, "that card represents my opinion of the danger which surrounds you, and if with that understanding you desire my services they are yours, but without that I refuse to serve you."

Dr. J. R. Corbus: I feel it my duty, as well as privilege, to state that I came from a neighboring city where diphtheria has been raging in an epidemic form for the past three years. It was my official duty as health officer to devise some means of checking the contagion, and the Mayor and City Council clothed me with power to use whatever means I saw fit, consistent with good judgment. The city of La Salle, where I came from, during the month of November two years ago had 95 cases of diphtheria and 35 deaths, out of a population of 10,000 inhabitants. There was no attempt made by the profession, or the former health officer, to prevent the intercourse of children and families, neither was there any attempt to placard the houses. I had an ordinance drawn up and passed, empowering me to placard the buildings, and to arrest anybody that was seen going into those houses after that notice was put up, and also to take immediate steps to isolate the patient, and, if necessary, to take charge of the patient and employ proper nurses. I carried these instructions out to the letter. The result of this procedure, with proper isolation and fumigation, lessened the disease the first month almost one-half, and the mortality in proportion That system of placarding and isolating has since been followed. Not only did I isolate, but I used prophylactic treatment. There were some instances where the families were poor (La Salle is a poor mining district), and these were taken care of. The result of this procedure is that the mortality is very much lessened, and the disease is almost under control, although it has been raging as a contagious disease in that city for the last three years. This ordinance was passed two years ago. At the next meeting of this society I will present the official report. I have it printed; also letters from physicians, calling my attention to the disease in families. The city of La Salle is composed of miners, who are dependent upon their daily labor, and when a family becomes stricken by disease, they have to have municipal help, and in that way I think it cost our city several thousand dollars in two years to support those people and give them proper treatment. In any instance where I thought the individual should remain in, I kept the whole family from mixing with their neighbors. Some of them thought it was pretty hard treatment, but it became a necessity, and they readily complied.

Dr. J. G. Kiernan: In regard to the contagion of diphtheria, while I do not lay claim to extensive experience, still there have been one or two instances under my direct observation that has raised the question in my mind whether it is not as desirable to isolate every case of diphtheria as rigidly as every case of scarlet fever. One instance I remember distinctly: A little girl had a favorite cat which she was in the habit of fondling. This occurred in a very healthy country neighborhood. The girl was taken sick with diphtheria; the cat was fondled for awhile, when the child maltreated it, and it ran away and made its appearance in a house two or three blocks distant. This child had contracted diphtheria in another place and brought it there. That house and the house where the cat took refuge were the only two houses in that village that had diphtheria. The people got scared at the cry of diphtheria and forced a quarantine. This instance proves to my mind that through the medium of domestic animals diphtheria can be conveyed. Such instances are frequent in the literature. During 1885 there was a diphtheria epidemic in Greece, conveyed by means of domestic fowls; and another conveyed by turkeys, in Asia Minor. Whether these are reliable is a question, but still the appearance, from time to time, in the literature, of cases of diphtheria conveyed by animals enforces a caution.

Dr. C. T. Parkes reported a case of

AMPUTATION OF THE SHOULDER FOR SARCOMA.

Professor Adelmann gave an address before the Surgical Society of Berlin, on the 4th of June last, concerning the operations for removal of the upper extremity, together with the scapula, a part or whole of the clavicle. His address contains the history of the operation, placing the date of the first reported case at 1808. The operation was next performed between 1830 and 1840 five times, between 1840 and 1850 five times, during the next decade three times, during the next seventeen times, during the next thirteen times, and since 1880 twenty-six times, making in all seventy reported cases. He discusses the statistics of Paul Berger, comprising fifty-one cases, and his method of operation. Adelmann makes three classes: (1.) Cases in which the operation was performed after traumatism. (2.) Those in which the operation was performed for benignant tumors. (3.) Those in which the operation was performed for malignant tumors. In the first class there are fourteen cases, with nine recoveries; in the second class three cases, with three recoveries; in the third class fifty cases, with twenty-four recoveries. These are subdivided into sarcomita, of which there were twenty-six, enchondromita, seven, encephaloid tumors, of which there were four, the remaining number bearing different names in different languages.

Of the fifty cases operated for malignant tumors, in twenty-five the entire operation was completed at one sitting. Among these cases there were ten recoveries. Of the twenty-five cases having more than one operation each, nineteen cases were operated in two sittings, with ten recoveries. Four cases had three operations each, with three recoveries. Of two cases with six operations each, one recovered. These recoveries, however, simply apply to the operation itself. Deaths from recurrence after healing of the wound are not counted in the statistics. Among the twenty-five cases in whom several operations were performed, there are seventeen in whom the arm was primarily removed; but having recurrence it was found necessary to remove the scapula and clavicle. Prof. A. remarks that this should induce us in the future to perform the entire operation at first; because these cases were all seen early, and the chances for radical cure must necessarily have been good; as it was, only five of all these twenty-five cases remained free from recurrence in after years. One of these after thirty years; another after twenty, two after six and one after three. In the fifteen cases of death after one operation, seven cases were due to the operation or to the low condition of tne patient at the time of operation. Two to shock, three to hæmorrhage, one to gangrene of the flaps, one tc purulent pleuritis and one to secondary hæmorrhage. In eight further cases in which the wound was completely or almost completely healed, the patient died from recurrence; this occurring five times in the lungs, the time of recurrence varying from three years to four months after the operation. In view of the frequent occurrence of secondary tumors in the lungs, the author advises careful examination of this organ before the operation, and considers the evidence of the presence of tumors in the lungs as a contra-indication for operation. The percentage of recoveries from this operation for malignant in all the reported cases, is a little less than fifty per cent. Many methods of operation have been adopted by the different operators, but the plan of ligating both the sub-clavian artery and vein primarily, seems to be advisable.

I will show the case as rapidly as possible, in order to let the patient get out of the room. You see that the wound is almost healed, all except this one spot of granulation. The boy, from his general appearance, is much healthier and stronger in every way than previous to the operation. You will notice that there are quite a number of little pleats here, as if the sewing had not been done very well; there is, apparently, a superabundance of flap at the upper part, which might have been used to close this gap of ulceration, but this resulted because I had not any plan in view before the operation, and made my flaps a little too redundant, so that when the lower flap was brought in contact with the upper one, its fullness caused the foldings during apposition.

This case came before the clinic at Rush Medical College: A boy, quite reduced from pain, displaying merely an enlargement of the upper end of the humerus, implicating the shoulder joint. The growth surrounded the bone, but was not uniform in development. Manipulation showed seeming fluctuation, both on the anterior and posterior aspect of the tumor; so much so, that friends who sent him supposed that all that would be necessary would be to open an abscess. But the general aspect of the patient, and the general appearance of the tumor, rendered me suspicious of it, and, being suspicious as to its nature, I introduced an exploring needle in order to ascertain whether it contained pus. Instead of getting pus I got only blood. The exploring needle went through the soft tissues to the bone, calling attention to the fact that there was not only implication of the soft parts, but as well disease of the bone itself. The fact seemed apparent that it was a case of sarcoma, which had attacked the shoulder joint itself, probably commencing in the capsule and passing from it to the tissues around it, and that it would be very likely to recur after amputation, or other simpler operation upon the shoulder joint. I put the case to the father, telling him that, as it was a malignant tumor, the only thing that seemed to me feasible was to do the operation of complete removal of the shoulder. He consented, and so the operation was done.

The immediate danger of the operation is hæmorrhage. There is another danger: the introduction of air into the veins when they are divided. All operations about the large vessels of the neck, or axillary space where the veins are apt to be patulous, are always a source of anxiety to the surgeon from their course. So, in order to overcome these immediate dangers, the thing to do primarily, before any attempt to make the incision for amputation, is to prevent the loss of blood directly and indirectly, by controlling the arterial and venous circulation, by ligating the subclavian artery and vein. These veins contain a large mass of blood, and if divided without control of them a good deal of blood would be lost, aside from the danger of the introduction of air into the vein.

Not having seen the reports of Paul Berger's method, I proceeded with the idea of controlling the hæmorrhage primarily. The first operation was done by an incision made above the clavicle, necessary for uncovering the subclavian artery, which was found and ligated close up to the side of the scalenus anticus muscle. The incision was then carried directly above to the top of the shoulder, the same as for amputation at the shoulder joint. This incision was prolonged in this way to the axillary space, and along the line of the axillary border of the scapula. As soon as the axilla was opened, the pectoralis major and minor muscles were divided, and instead of tying immediately the axillary vein, it was included between two hæmostatic forceps, and the vein divided between them; then the main trunks of the brachial plexus were divided. Then the arm was drawn over the front of the body, and this incision adopted for the excision of the scapula, running along the line of the scapula so that the posterior flap was divided into two portions, and these two flaps were rapidly dissected off, until the posterior part of the scapula was uncovered, it raised from the chest wall, and the muscles divided, and the extremity removed. All bleeding points, with axillary vein, were now ligated, and the flaps united. This operation was not done upon any specific plan. Following the suggestion of Mr. May, who, in the December issue of the *Annals of Surgery*, reports two cases of this operation, I have looked through all the books of my library, but have not found any specific method given for this operation. It remained for Paul Berger to give a plan for it. He was led to the plan he suggests after several trials on the cadaver. The quickest and easiest way of doing the operation and securing the blood-vessels is his plan of procedure. His plan is to make the incision from the inner extremity of the clavicle outward to the top of the shoulder, and then immediately uncover the clavicle and turn it out of the way; that leaves the subclavian vessels exposed, so that they can be easily secured. You all remember well, as the result of your past experience, that as one uncovers the front of the axillary space, there is always to be seen a ridge across it, produced by the raising of loose tissue and upon the external anterior thoracic nerve. It is easily found, and the reason I call attention to it is that as you pass outward along this nerve, it leads directly to the interval between the artery and vein, with the clavicle out of the way; and hence to them the vessels are superficially situated, easily isolated, and also free from diverging branches. The artery should be tied in two places an inch apart, and divided, and the vein also; then the circulation is absolutely under control. May advises that just before the vein is tied the arm should be elevated and held up a few minutes, in order to let the venous blood drain out of it, thus saving as much as possible of the blood for the individual. In my second case I applied the Esmarch bandage up to the axilla, squeezing the blood out of the veins entirely; then, as soon as the arteries are secured in this position, with a rapid cut of the scissors the brachial plexus can be divided, and the pectoralis minor and major severed. The flap portion of the operation is done in this way: Commence at the center of the anterior incision, and carry the knife directly across the anterior part of the axilla to the lower angle of the scapula; then commence at the outer edge of the incision posteriorly, and carry the knife behind the joint to the same point; rapidly reflect the posterior flap; then all the muscular attachments should be divided, and the extremity removed without any trouble. This gives a perfectly even anterior and posterior flap, coming together easily and nicely, and one avoids the unseemly appearance of the anterior part of this wound, which was occasioned by the anterior flap being too redundant.

This operation was done six weeks ago, and there has been no time since the operation when we felt particularly anxious about the patient's recovery except the first few days. The patient's perfect recovery has been interfered with from an accident, the effects of which you notice, the sloughing of the flaps, leaving this ulcer. In dissecting up the flaps one is compelled to keep close to the skin, diminishing greatly the nourishment of the immense piece of skin. The danger is increased if one is not careful to avoid wounding the post-scapular artery; so it is necessary to bear in mind the direction of these incisions in order to secure as nice a stump as possible.

The second case came in about two weeks after this, demonstrating the assertion that almost all cases come in couples. This case was a man 37 years old, who came in one afternoon with a tumor on the top of his shoulder, occupying the situation of the supra-spinous fossa. It had all the indications, so far as external appearances went, of a fatty tumor. Those who have charge of clinics labor under this disadvantage in all their cases. They do not have the opportunity of examining them previously, as surgeons in hospitals do, hence they are apt to go into a case without as complete an examination as the case is entitled to. This was examined hastily and the history hastily passed over, and the suggestion made that in all probability it was not a fatty tumor; and from the rapidity of its growth would prove to be malignant, and one that was connected with the superficial tissues of the spinous fossa. But as soon as the incision was made and it was exposed we saw the mistake, because it proved to be a tumor that grew primarily from the shoulder joint, and particularly to some part of the capsular ligament crowding out from beneath the supra-spinous fossa, and developing as large as a cocoanut upon the man's shoulder. Previous to his going to sleep no consent had been obtained to do so radical an operation as entire ablation of the upper extremity, so only a temporizing operation was done, the removal of the tumor so far as external manifestations were concerned. After he came out from the ether and inquired about it and had the nature of the growth explained to him, after consulting with his friends, in about three weeks he decided to have this operation done, and it was done, but he died in fifty-six hours after the day of operation. He was slightly shocked by the operation but recovered from that, and for twenty-four hours was quite well, with only slight elevation of temperature and pulse; then he was taken with delirium and died in a comatose state. I do not know exactly what was the cause of death, but I am inclined to think that it was poor policy to do this severe operation so soon after the primary interference. The man was still depressed, and in great fear of the severity of this operation. All these facts were against him. In this case, with the operation by the method I have described as advocated by Paul Berger, I am quite sure it was more quickly done and with more satisfaction to the operator, and if he had lived, to the patient.

This second case properly comes under the head of secondary operations. It is quite noticeable from the report read, that the cases done by machinery are all reported as recoveries, and it is questionable whether they have a place at all in the classification of this operation, because the cause of death after the accident is not reported at all.

